# Traditional and photocatalytic conversion of aniline into azocompounds – a comprehensive experimental and theoretical review

**DOI:** 10.1039/d5ra03828f

**Published:** 2025-08-08

**Authors:** Afsar Khan, Hong Ran, Chen Saisai, Nisar Muhammad, Adnan Abbas, Zhang Yi, Faheem Shah, Weihua Chen, Dayong Xu

**Affiliations:** a School of Chemical and Environmental Engineering, Anhui Polytechnic University Wuhu 241000 China xdy826@ahpu.edu.cn; b Department of Chemistry, College of Science, King Faisal University Hofuf Eastern Province AlAhsa-31982 Kingdom of Saudi Arabia fshah@kfu.edu.sa; c School of Chemistry and Environmental Science, Shangrao Normal University Shangrao 334000 China chenweihua419818@sina.com

## Abstract

The catalytic conversion of aniline into azo compounds represents a significant and versatile route in synthetic organic chemistry, with applications spanning dyes, pharmaceuticals, and functional materials. This comprehensive review systematically examines recent advances in catalytic strategies for azo bond formation from aniline derivatives, encompassing homogeneous, heterogeneous, enzymatic, and photo-redox catalysis. Key mechanistic pathways, including oxidative coupling, dehydrogenative aromatization, and redox processes, are critically analyzed. The influence of catalyst design (*e.g.*, transition metals, organo-catalysts, nanomaterials) and reaction conditions (solvent, oxidants, temperature) on selectivity and efficiency is discussed. Additionally, the review highlights sustainable approaches, such as aerobic oxidation and visible-light-driven catalysis, aligning with green chemistry principles. Challenges, including substrate scope limitations and scalability, are addressed, along with emerging trends and future prospects for industrial and academic applications. This work aims to serve as a foundational resource for researchers exploring catalytic azo compound synthesis.

## Abstract 2

The catalytic conversion of aniline into azo compounds represents a significant and versatile route in synthetic organic chemistry, with applications spanning dyes, pharmaceuticals, and functional materials.

## Graphical abstract

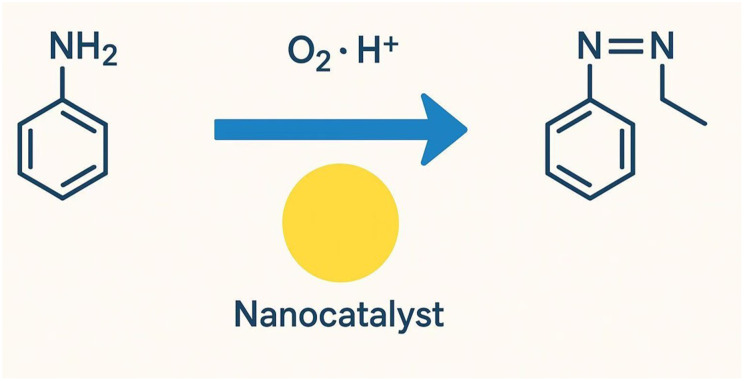

## 1 Introduction

Aromatic azo compounds are a significant class of molecules featuring the –N=N– chromophore and extended conjugated systems. This structural characteristic grants them diverse applications in various fields, including radical reaction initiators, organic dyes, photosensitive materials, pigments, and indicators. Additionally, aromatic azo compounds serve as crucial intermediates in numerous organic conversion processes.^1–4^ Traditional methods for synthesizing aromatic azo compounds typically involve diazonium coupling or Mills reaction. The diazonium coupling reaction offers advantages such as rapid reaction rates and high yields. However, it imposes significant limitations on the types of substituents allowed on the aromatic rings, thereby restricting the substrate scope. The Mills reaction typically requires acetic acid as a catalyst and involves nitroso aromatic compounds and primary aromatic amines as starting materials. However, the instability of nitroso compounds often leads to side reactions. Over the past decade, significant efforts have been made to develop new catalytic methods for synthesizing aromatic azo compounds through the direct oxidative coupling of anilines.^1,5–7^

Transition metal catalysts^8^ offer a promising option for industrial-scale production of azo compounds due to their low cost and high efficiency.^9,10^ Furthermore, various strategies, including element doping, structural recombination, crystal plane orientation growth and morphology regulation, can be employed to optimize these catalysts for azo compounds synthesis.^11,12^ Metal nanoparticles and metal supported nanoparticles effectively used for azo compounds synthesis.^2^ To enhance catalytic efficiency, researchers have developed sophisticated design strategies for metal nano particles, focusing on controlling particle size and shape, modifying electronic properties, and optimizing metal–support interactions.^13,14^ The support material significantly influences the catalytic performance of metal nanoparticles for azo compounds synthesis, particularly at the metal–support interface where active sites are located.^4^ However, these active sites are relatively few, limiting overall catalytic activity. Furthermore, the metal–support interaction can weaken during reactions, leading to aggregation of active metal species and reduced performance.^12^

Research has shown that incorporating alkali metals into catalysts can significantly enhance their electron transfer properties, leading to improved catalytic conversion of aromatic amines into azo compounds.^15^ Alkali metals offer advantages over transition metals, including higher charge density, stronger bonding with functional groups, and being lightweight and non-toxic. These benefits make alkali metal incorporation an attractive approach for enhancing catalytic performance.^16^

Gold catalysts have recently gained significant attention in organic synthesis due to their versatility and effectiveness. Recent studies, particularly those focusing on gold–support interactions, have enabled practical applications of gold catalysts.^6^ Notably, Grirrane *et al.*^6^ reported that Au/TiO_2_ and Au/CeO_2_ nanocatalysts demonstrated high conversion and selectivity for aniline to azo compound synthesis using O_2_ as the oxidant. Zhu *et al.*^12^ demonstrated the use of Au/ZrO_2_ catalyst in a photocatalytic reaction, achieving high conversion of nitro compounds under atmospheric pressure. Research has shown that the performance of gold catalysts can be enhanced by selecting suitable supports and controlling gold nanoparticle size. Despite this progress, achieving both high conversion and high selectivity in the oxidation of aniline to azoxy compounds remains a significant challenge in supported metal catalysis systems. Li *et al.*^17^ found that modifying ZrO_2_ with Fe altered its crystal structure and increased the number of surface acidic and basic sites, enhancing the activation of methanol and CO_2_. Similarly, introducing Fe into Cu-based catalysts for dimethyl carbonate formation improved catalytic activity due to a balanced distribution of acidic and basic sites, attributed to the combined effects of metallic Cu, Fe species, and oxygen-deficient Fe_2_O_3_.

## 2 Methods of synthesis of azo compounds

### 2.1. Direct oxidation of aromatic amines and their derivatives

**Fig. 1:**
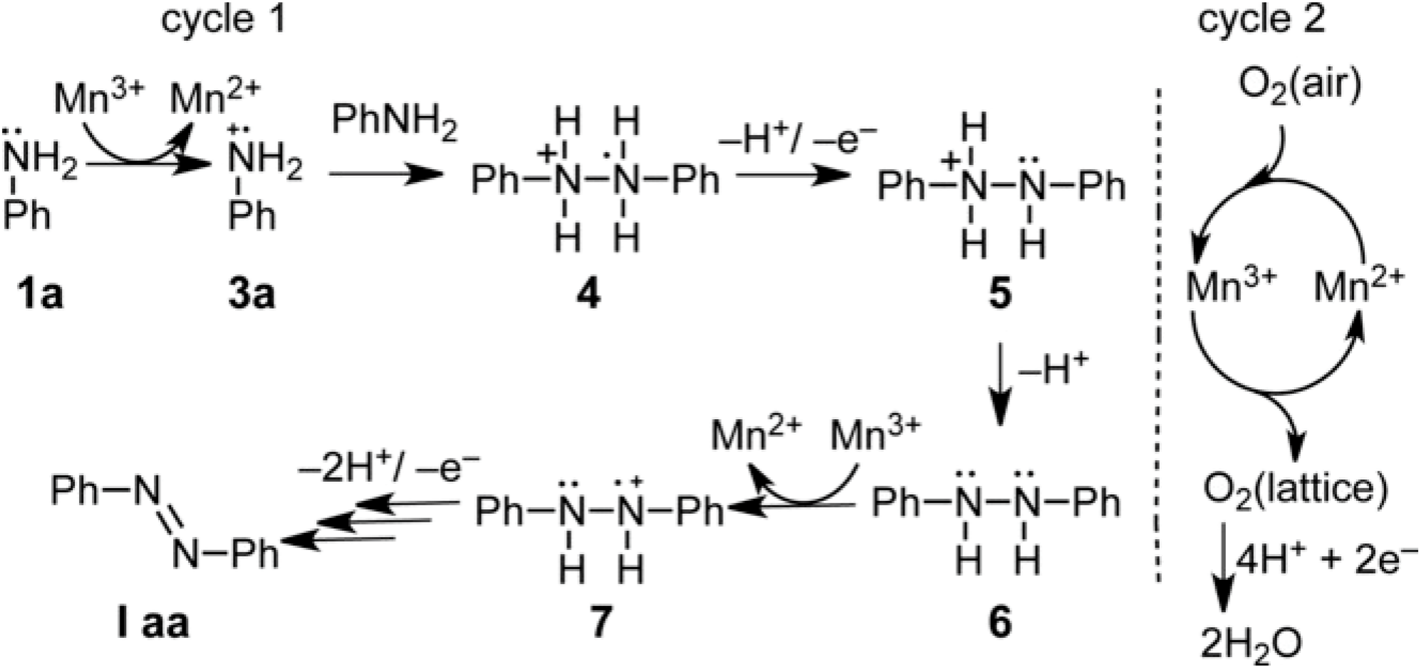
Mesoporous manganese oxide catalyzed aerobic oxidative coupling of anilines to aromatic azo compounds.^18^

The direct oxidation of aromatic amines to azo compounds is a promising and environmentally friendly method. Oxygen is the ideal oxidant due to its green and atomically economical nature. However, oxygen molecules typically require catalytic activation to participate in the reaction, limiting their direct use. Transition metal compounds have gained attention for variable oxidation states and redox potentials, enabling catalytic activity. Additionally, these catalysts can be regenerated using oxygen from the air, making the process more sustainable. Dutta *et al.*^18^ devised an economical approach for synthesizing aromatic azo compounds, employing a mesoporous manganese oxide catalyst with air serving as the oxidant. This approach enabled the formation of diverse unsymmetrical and symmetrical azo compounds from aniline derivatives under mild atmospheric conditions, with moderate to excellent yields. The catalyst showed good reusability, making the protocol promising for practical applications. They assumed that the radical species 3a formed from aniline (1a) was coupling with another molecule of aniline 1a to form 4 (a compound with a 3e^−^ σ bond), which through the successive loss of a proton, an electron, and another proton formed intermediate 6. Intermediate 6 goes through all the aforementioned steps once again to provide the final product Iaa^18^ (Fig. 1).

**Fig. 2:**
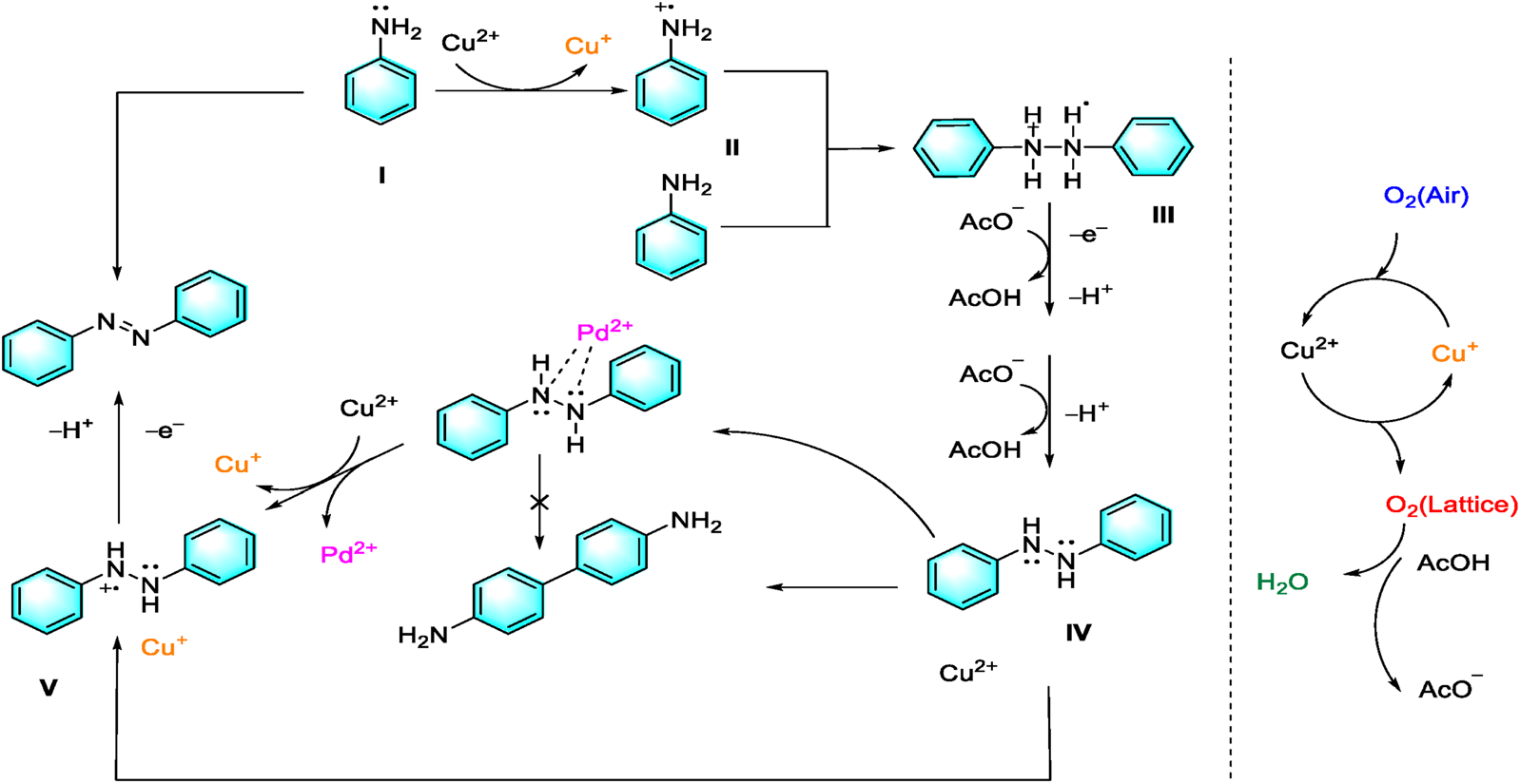
Oxidation coupling of aniline to form azo compounds.^19^

The widespread use of solvents in organic synthesis contributes to environmental issues, making solvent-free reactions a desirable goal. Researchers have developed a method using copper acetate and a trace amount of palladium salt enables the efficient conversion of anilines to aromatic azo compounds under base- and solvent-free conditions. This approach also achieved satisfactory yields in cross-coupling reactions.^19^ Copper played a fundamental role in the catalytic process, aided by palladium salt and oxygen. This method offers a new way for synthesizing asymmetric and symmetric azo compounds and highlights the potential of transition metal compounds in catalysis (Fig. 2).

Maier *et al.*^20^ developed a new method for synthesizing cyclic azo benzenes from diarylamine precursors, overcoming challenges related to steric hindrance and angular tension. The reaction utilized *m*-CPBA as the oxidant in a mixed solvent system of acetic acid and dichloromethane. The synthesis of cyclic azo-benzenes offers a new type of photo-molecular switch. However, the method has limitations, including reduced yields for diarylamines with strong electron-withdrawing or electron-donating groups, and the need for additional derivatization steps for amino-substituted diarylamines. Furthermore, the reaction can generate multiple by-products and shows limited substrate selectivity, with substituent electronic properties having a weak impact on product yields. Direct oxidation of aromatic amines to form azo compounds necessitates precise control of the oxidation level to minimize the formation of by-products such as nitrobenzene and azobenzene oxide.

Shukla *et al.*^21^ developed a Cu–CeO_2_ nanoparticle catalyst that achieved high yields and selectivity for azo compounds using H_2_O_2_ as the oxidant and acetonitrile as the solvent. Under optimized conditions, the catalyst delivered 95% aniline conversion and 92% azobenzene selectivity. Notably, the catalyst showed stability and heterogeneity, with minimal changes in Cu and Ce content after use. While significant progress has been made in synthesizing aromatic azo compounds, constructing heteroaromatic azo derivatives remains underexplored.

Jiang *et al.*^22^ introduced a novel method for synthesizing azo compounds through iodination of pyrazol-5-amine, utilizing *t*-butyl hydroperoxide (TBHP) as the oxidant and a copper salt as the catalyst. This approach enables the simultaneous formation of C–I and N–N bonds, followed by oxidative dehydrogenation to produce azopyrroles and iodo-substituted azopyrroles with diverse substituents. The reaction conditions are mild, and the catalytic system allows for selective modification of the pyrrole skeleton. However, the low yield in derivatization reactions needs optimization for practical applications. The direct oxidation of aromatic amines to synthesize azo compounds offers several advantages, but over-oxidation can lead to the formation of unwanted by-products.

### 2.2. Reductive coupling of aromatic nitro compound

Aromatic nitro compounds can be reduced to aromatic amines, making them a potential starting point for synthesizing azo compounds. A one-step synthesis from nitro compound to azo compound would enhance the economy and environmental sustainability of the reaction. This approach has gained significant attention in the scientific community.

Mondal *et al.*^23^ developed a method to prepare Au nanoparticles using a nanoscale organic cage as a template, contributing to this area of research. The cage-immobilized Au nanoparticles serve as a heterogeneous catalyst for selectively reducing nitroaromatics to azo compounds using 2-propanol, achieving high yields at room temperature. Under optimized conditions, 99% conversion to the corresponding azo compounds was obtained after 2 hours of UV irradiation in an inert atmosphere. Notably, no reaction occurred without Au nanoparticles, highlighting their crucial role in the process.

Yan *et al.*^24^ developed a method where Pd(OAc)_2_ was reduced *in situ* by NaBH_4_ to form ultra-fine Pd nanoclusters (PdNCs), which were stabilized by nitroaromatics and dispersed in solvent. These PdNCs catalyzed the selective reduction of nitroarenes to various products with high yields, including those with electron-donating and electron-withdrawing groups. The protocol also worked well for nitro-fused aromatic compounds like nitronaphthalene, achieving an 80% yield. However, the reaction's selectivity needs improvement due to the potential oxidation of hydroxylamine, which can lead to side reactions. Further optimization of reaction conditions is necessary.

**Fig. 3:**
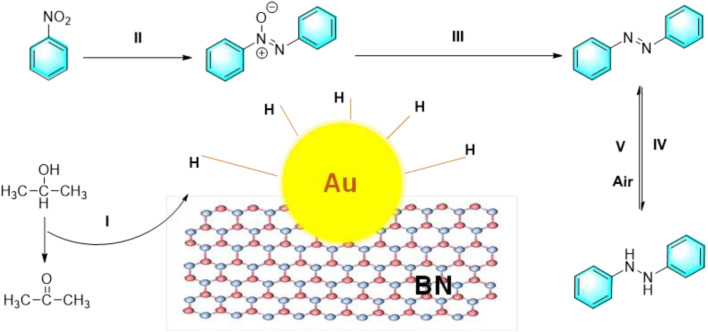
The reduction of nitrobenzene catalyzed by Au/BN.^25^

Hexagonal boron nitride is a 2D material with thermal stability and low toxicity making it suitable for catalytic applications (Fig. 3). It provides a platform for metal nanoparticles, offering more reactive sites. Research has shown that h-BN can inhibit oxygen activation, allowing catalytic hydrogenation of nitrobenzene in air. In a reaction mechanism, KOH facilitates hydrogen transfer from isopropanol to the Au/BN surface, forming an activated H donor. The H donor binds to Au nanoparticles, creating H–Au, which plays a crucial role in the reduction process. The H–Au species can also release H_2_, enabling a continuous cycle.^25^

### 2.3. Photocatalytic method

**Fig. 4:**
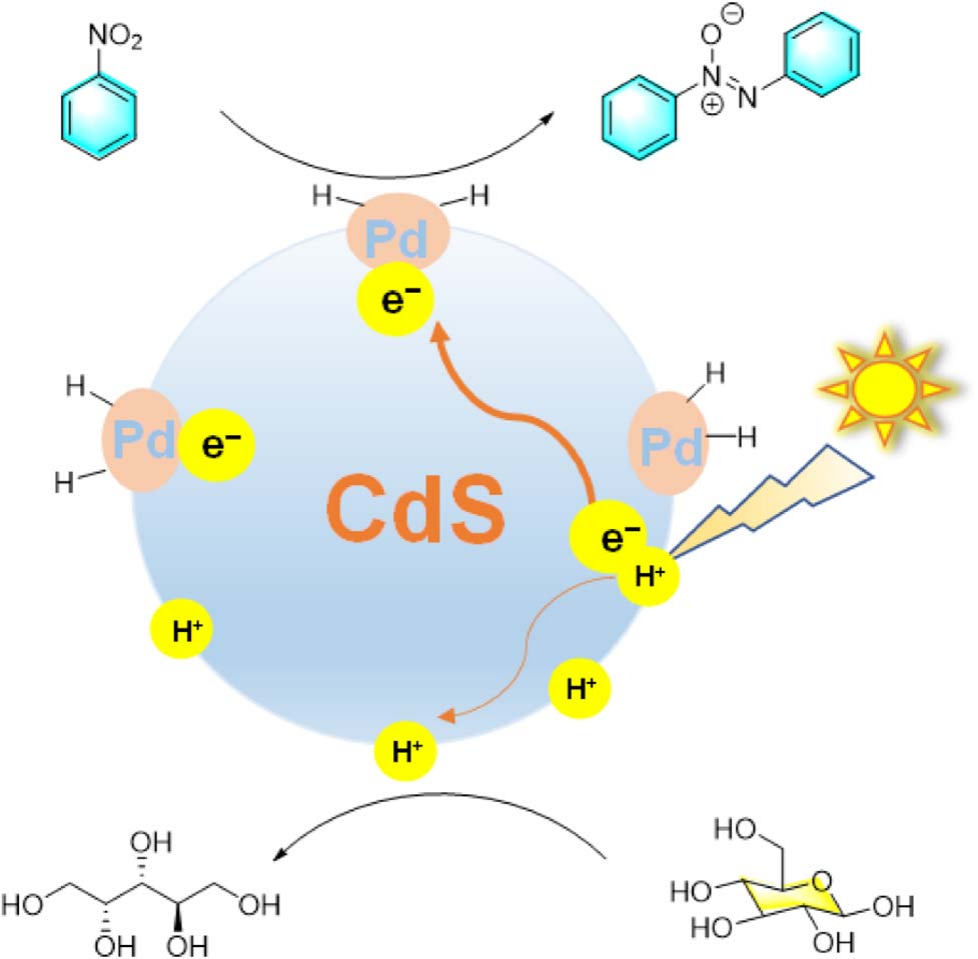
Photochemical reduction of nitrobenzene catalyzed by Pd@CdS.^26^

Photocatalytic synthesis is a promising method in organic chemistry that utilizes light energy to drive reactions. It offers advantages such as fast reaction rates, high selectivity, mild conditions, and reduced energy consumption compared to traditional methods. Zhou *et al.*^26^ demonstrated that Pd nanoparticles loaded on mesoporous CdS (Pd@CdS) serve as a highly active and selective photocatalyst in water under visible light irradiation (Fig. 4). The composite catalyst Pd@CdS enables simultaneous photocatalytic conversion of glucose to arabinose and nitrosobenzene to azobenzene with high selectivity. Under visible light irradiation, photoexcited electrons are transferred from CdS to Pd nanoparticles, inhibiting electron–hole recombination and facilitating the reduction of nitrosobenzene to azobenzene. Meanwhile, glucose is oxidized by holes to form arabinose. The process is further enhanced by the interplay between the two reactions, where formic acid generated from glucose oxidation aids the hydrogenation of nitrobenzene, and nitrobenzene promotes glucose conversion by accepting electrons and H^+^ ions.

Wang *et al.*^27^ developed a CQDs-ZnIn_2_S_4_ nano-catalyst for the controlled catalytic hydrogenation of nitrobenzene under visible light. By adjusting the hydrogen source, and alkalinity the reaction can selectively produce azobenzene, azoxybenzene, or aniline. The mechanism involves ZnIn_2_S_4_ generating electrons and holes under light irradiation, with CQDs transferring electrons to facilitate the hydrogenation process. The addition of bases like NaOH promotes the formation of azobenzene or azoxybenzene. While photocatalytic synthesis offers many benefits, it still faces challenges like limited solar energy utilization, low quantum efficiency, and inadequate catalyst recyclability.

**Fig. 5:**
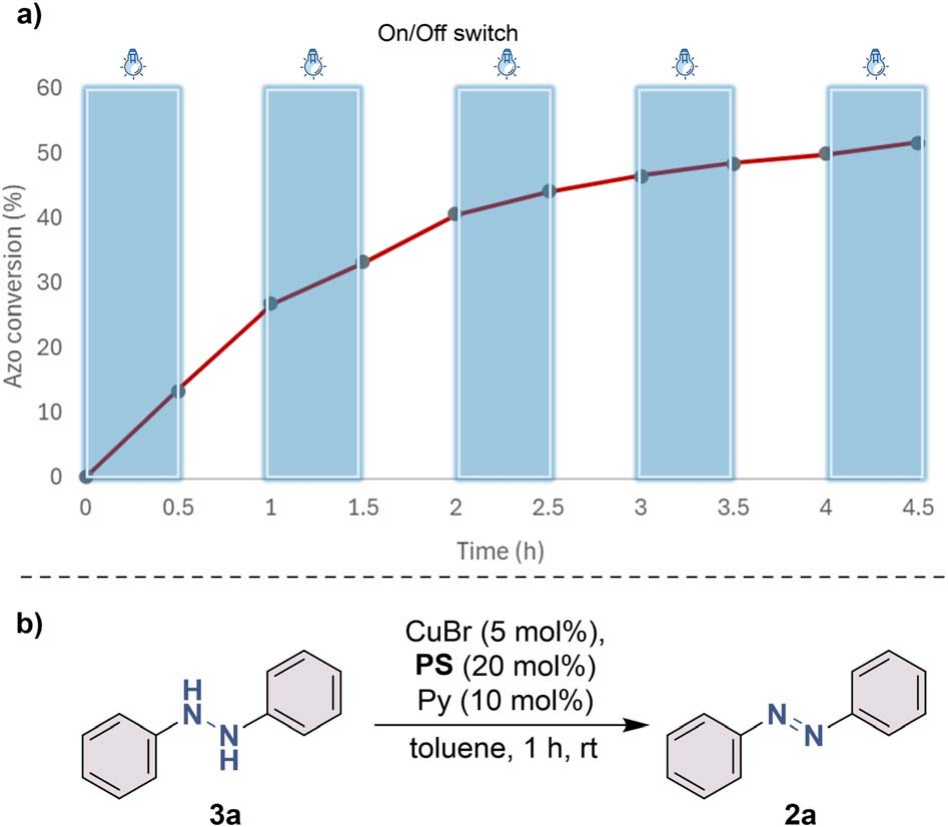
(a) Light on/off experiment to track chain propagation. Blue columns represent the time period in which the light was on. The conversion data points were recorded using ^1^H NMR in the span of 4.5 hours. (b) Reaction was run using hydrazine instead of aniline as the starting reagent in the absence of blue light.^28^

Rowshanpour *et al.*^28^ reported the first instance of photo-dual catalytic Cu/photosensitizer synthesis of symmetric and non-symmetric azo compounds from readily available amines using renewable light energy input, ambient conditions, and ubiquitous air as a stoichiometric oxidant (Fig. 5).

### 2.4. Aniline conversion by various catalytic materials

Lima *et al.*^29^ reported the selective conversion of aniline to azoxybenzene at room temperature using amorphous niobium oxyhydroxide supported on δ-FeOOH. The catalysts were synthesized *via* a co-precipitation method by incorporating varying amounts of niobium oxyhydroxide into δ-FeOOH nanoparticles. This modification altered both the chemical and textural properties of δ-FeOOH, leading to a marked improvement in its catalytic efficiency for aniline oxidation in the presence of hydrogen peroxide. The catalytic activity and selectivity were influenced by the niobium content in the composite. The study examined the impact of various reaction parameters, such as solvent type, hydrogen peroxide volume, and reaction time. Among the tested catalysts, the one containing 10 wt% Nb exhibited the highest performance, achieving complete aniline conversion and 80.2% selectivity to azoxybenzene when propanol was used as the solvent at 25 °C. Additionally, this catalyst showed excellent stability and reusability, retaining its activity across multiple reaction cycles.

Jiang *et al.*^19^ developed a straightforward approach for synthesizing both symmetric and asymmetric aromatic azo compounds directly from anilines, employing Cu(OAc)_2_ as a catalyst under base-free and solvent-free conditions. The introduction of a small amount of palladium salt further enhanced the reaction yield. This approach also showed promise for cross-coupling nitridation reactions.

Wang *et al.*^30^ developed a method for synthesizing azo-benzenes through the oxidative coupling of anilines using transition metal oxides, with manganese oxide OMS-2 showing the best activity and selectivity. They synthesized various symmetric and unsymmetric azo-benzenes with high conversion and selectivity rates. The selectivity of unsymmetric azo-benzenes was influenced by the difference in Hammett constants between the substituent groups. Research suggested that surface defect sites on the manganese oxide facilitate electron transfer and oxygen activation, proposing a single-electron transfer reaction mechanism based on detailed characterization.

Patel *et al.*^31^ reported a method using a polyoxometalate-based molecular molybdenum oxide catalyst to oxidize anilines to azobenzene and azoxybenzene compounds. This approach is compatible with a wide range of substrates and functional groups, allowing for the efficient synthesis of symmetric and asymmetric azo-benzenes and azoxybenzenes using H_2_O_2_ as a green oxidant. Mechanistic studies suggest the formation of highly active Mo imido complexes as intermediates.

**Fig. 6:**
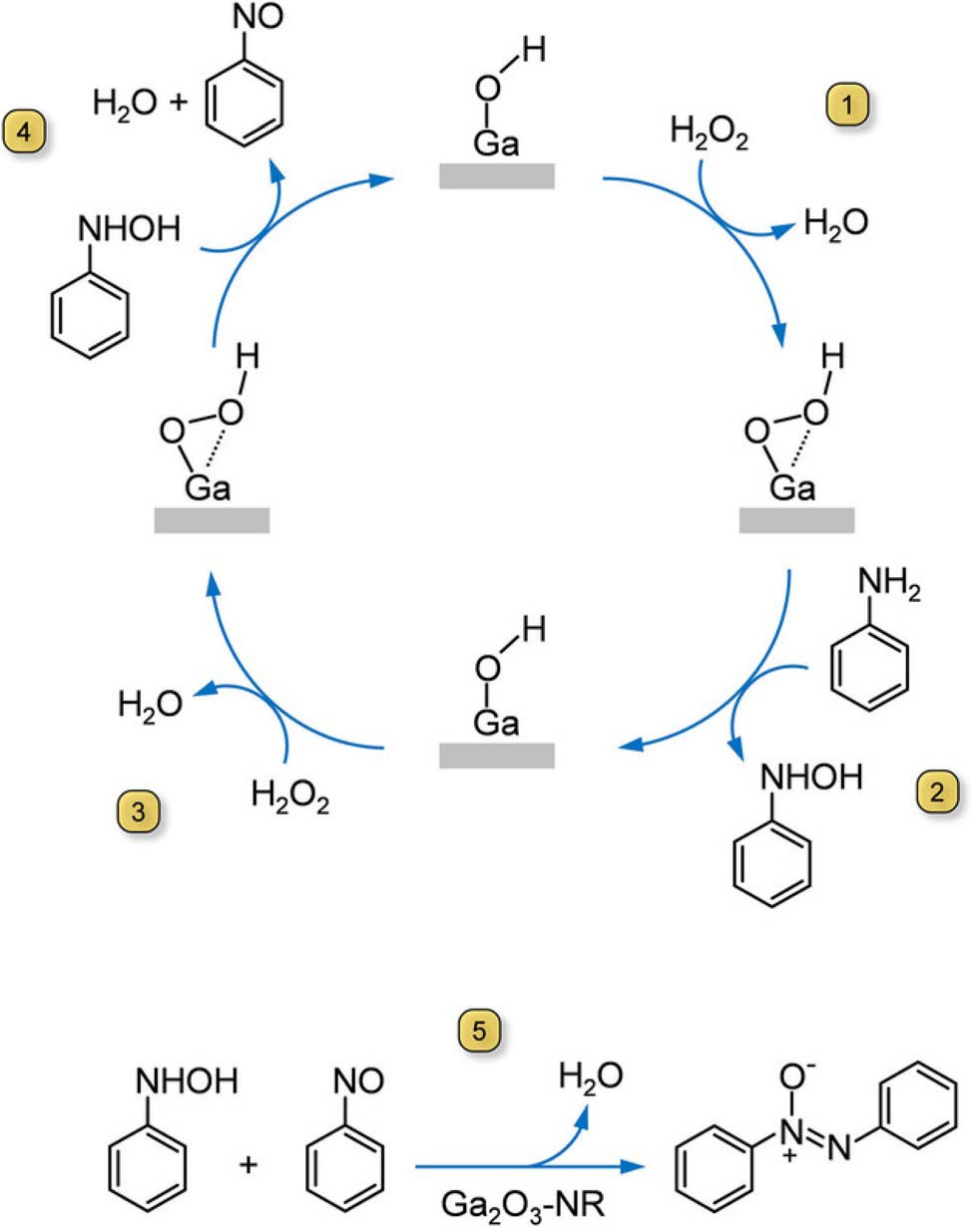
Mechanism of aniline conversion to azo compounds over aluminium and gallium oxide catalysts at various conditions.^3^

Singh *et al.*^3^ developed a method for selectively oxidizing aromatic amines to azoxy derivatives using aluminum and gallium oxide nanorod catalysts. Gallium oxide nanorods, with their small size and high surface area, showed exceptional performance, achieving 94% aniline conversion with 98% selectivity to azoxybenzene. Notably, this reaction used hydrogen peroxide as a green oxidant and did not require transition metal catalysts. The catalyst also demonstrated excellent reusability, retaining its activity and selectivity over multiple runs. The detail mechanism is given in Fig. 6.

Patel *et al.*^31^ reported a direct oxidative azo coupling of anilines using a self-assembled, flower-like CuCo_2_O_4_ catalyst under aerobic conditions. The catalyst was synthesized *via* the oxalate decomposition method and characterized using raman spectroscopy, powder X-ray diffraction, transmission electron microscopy (TEM), field-emission scanning electron microscopy (FESEM), and energy-dispersive X-ray spectroscopy (EDX). The flower-like CuCo_2_O_4_ demonstrated excellent catalytic performance for the direct aerobic oxidative azo coupling of anilines under additive- and oxidant-free conditions. Density functional theory (DFT) calculations provided mechanistic insights, revealing that atmospheric oxygen preferentially adsorbs and dissociates at the Cu sites, while aniline dissociation occurs at the Co sites. This cooperative interaction between Cu and Co sites facilitates the selective activation of both oxygen and aniline during the reaction. The catalyst was recyclable and maintained high catalytic activity over at least eight consecutive cycles.

Shukla *et al.*^21^ reported the catalytic oxidation of aromatic amines to azoxy compounds using a Cu–CeO_2_ catalyst and hydrogen peroxide (H_2_O_2_) as the oxidant. The catalyst was synthesized *via* a one-pot hydrothermal method employing cetyltrimethylammonium bromide (CTAB) as a surfactant, resulting in Cu nanoparticles supported on nanocrystalline CeO_2_. Characterization techniques including XRD, SEM, TEM, XPS, TPR, EXAFS, BET surface area analysis, and UV-vis spectroscopy confirmed the successful formation and morphology of the catalyst. The catalyst exhibited excellent activity and selectivity for the oxidation of aromatic amines to their corresponding *N*-oxides. Optimal performance was observed with a Cu loading of 3.8 wt%, which yielded 5–10 nm Cu nanoparticles supported on 20–40 nm CeO_2_ particles. At this loading, the catalyst achieved 95% aniline conversion and 92% selectivity for azoxybenzene. Higher Cu loadings led to agglomeration of Cu species, resulting in decreased catalytic efficiency. The study also systematically examined the influence of various reaction parameters, including temperature, reaction time, substrate type, and H_2_O_2_ molar ratio.

**Fig. 7:**
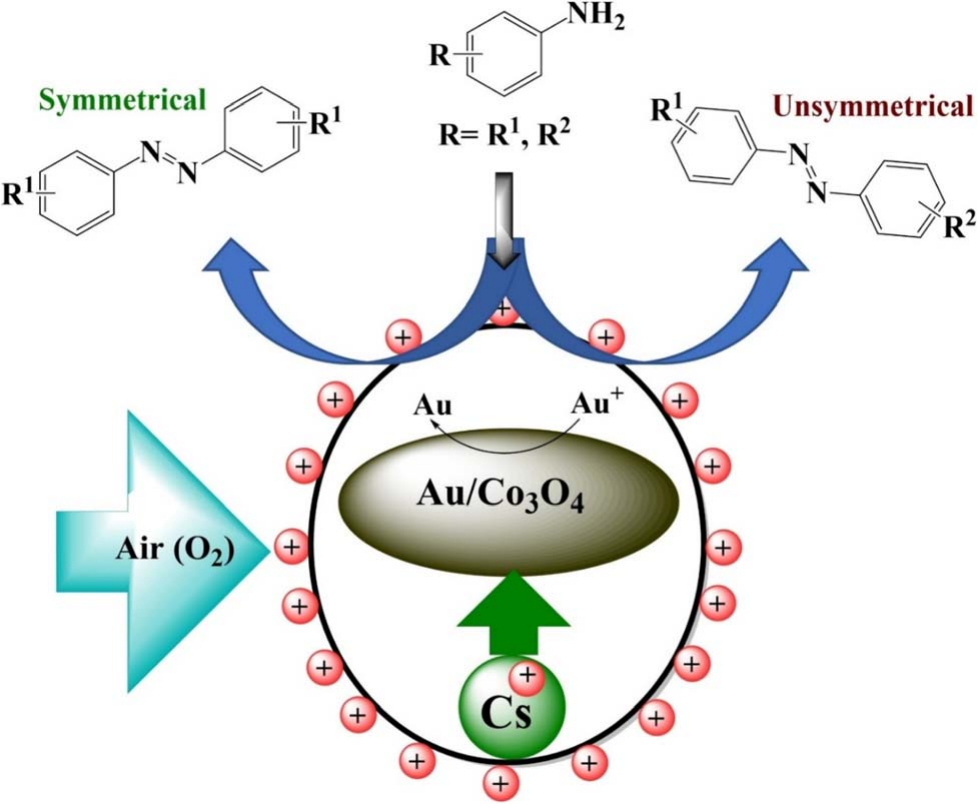
Mechanism involved oxidation of aniline into symmetrical and unsymmetrical azo compounds.^32^

Oseghale *et al.*^32^ reported the development of a bifunctional Cs–Au/Co_3_O_4_ catalyst, possessing both basic and redox properties, for the oxidative synthesis of aromatic azo compounds from anilines (Fig. 7). This eco-friendly, alkali-promoted catalyst enabled the oxidative dehydrogenative coupling of anilines to produce both symmetrical and unsymmetrical azo compounds using air as the oxidant, eliminating the need for base additives or toxic molecular oxygen. The catalyst demonstrated good reusability and operated efficiently under mild conditions. Enhanced catalytic performance was attributed to a higher concentration of basic sites, lower reduction in temperatures, and the active role of lattice oxygen in the nanostructure. The increased basicity promoted electron density on the active Au species, significantly boosting the yield of aromatic azo products.

Dutta *et al.*^18^ established a straightforward, economical, and mild catalytic method for synthesizing a broad spectrum of symmetrical and unsymmetrical aromatic azo compounds using inexpensive meso-MnO_*x*_ materials. A variety of aniline derivatives underwent both oxidative homocoupling and cross-coupling reactions, yielding products in moderate to excellent yields (35–99%). The use of air as the terminal oxidant, the absence of precious metals or additives, operation under ambient conditions, and good catalyst reusability highlight the advantages of this system over existing methods. Mechanistic studies revealed the key roles of surface-active Mn^3+^ species, labile lattice oxygen, and radical intermediates in the reaction cycle. A manganese-mediated electron-transfer mechanism was proposed and supported by density functional theory (DFT) calculations. Further studies are ongoing to refine the mechanism and enhance reaction efficiency.

**Fig. 8:**
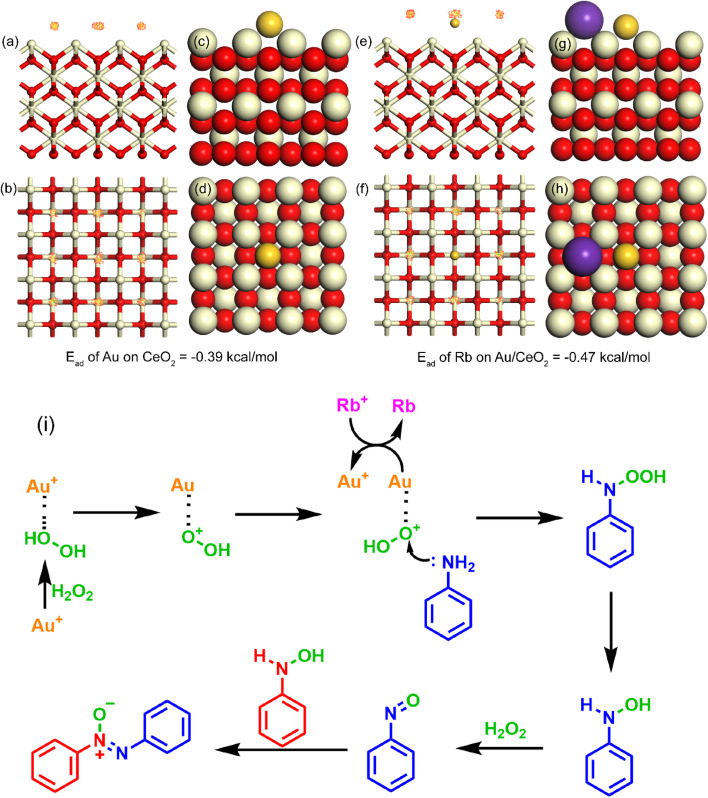
(a) Side view of Au/CeO_2_ (100) adsorption fields (b) top view of Au/CeO_2_ (100) adsorption fields (c) side view and (d) top view of Au/CeO_2_ (100) most stable adsorption site (e) side view and (f) top view of Rb doped Au/CeO_2_ (100) adsorption fields (g) side view and (h) top view of Rb doped Au/CeO_2_ (100) most stable adsorption site (i) proposed mechanism of the reaction.^1^

Khan *et al.*^1^ reported the development of a Rb-doped Au/CeO_2_ nanocatalyst that demonstrated exceptional efficiency for the selective oxidation of anilines to azocompounds using hydrogen peroxide as the oxidant. The catalyst achieved complete aniline conversion (100%) and remarkably high selectivity (92%) toward azoxybenzene. The incorporation of Rb^+^ was found to promote electron transfer, enhancing the formation of catalytically active Au^+^ species. A DFT calculation is done to probe in the exact interaction mechanism of the catalyst surface and product produced (Fig. 8). Table 1 shows various catalytic activities at various conditions.

**Table 1:** Comparison between activities of various reported catalysts at various conditions.^1^

Catalyst	Conversion (%)	Selectivity	Solvent	*T* (°C)	Time (h)	References
7.5% CuCeO_2_	82	72	CH_3_CN	50	6	7
Al_2_O_3_-nanopowder	14	38	EtOAc	80	4	3
Ga_2_O_3_-NR	49	92	EtOAc	80	4	3
Ag/Fe_2_O_3_ nanocatalyst	91	92	CH_3_CN	50	8	33
Nb_2_O_5_-scCO_2_	86	79	EtOH	25	1	34
Nb-doped iron oxides	33	59	EtOH	25	24	35
Al_2_O_3_/AlNbO_4_	80	82.7	DMF	25	24	36

**Fig. 9:**
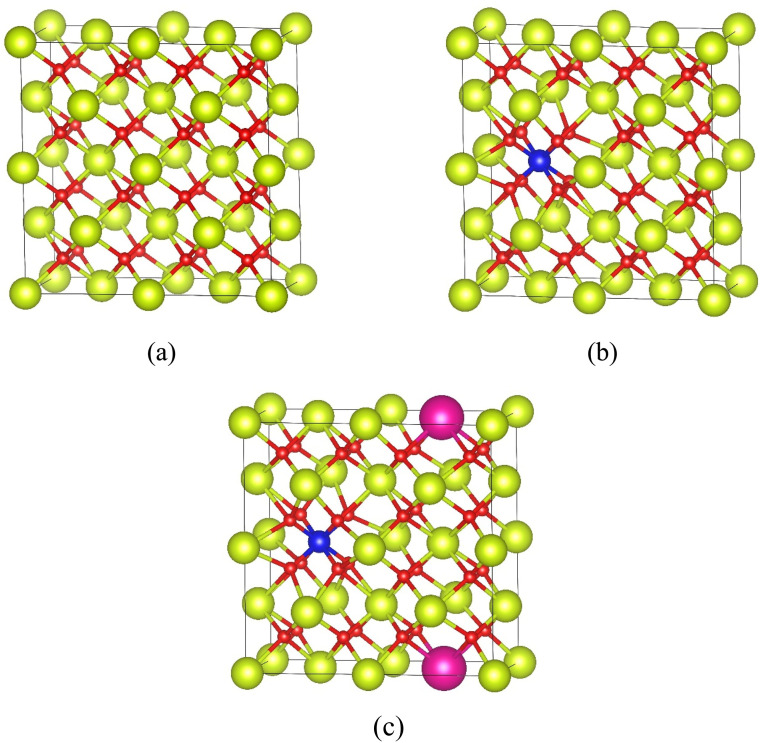
Optimized structures of the CeO_2_ (a), Fe/CeO_2_ (b), Rb- Fe/CeO_2_ (c) supercells. Red, yellow, blue, and fuchsia correspond to oxygen, cerium, iron, and rubidium atoms, respectively. The solid black line confines the supercell.^2^

Khan *et al.*^2^ also reported a Rb-promoted Fe/CeO_2_ nanocatalyst to investigate the role of Rb as a promoter in enhancing the catalytic performance for the selective oxidation of aniline to azoxybenzene using H_2_O_2_ as the oxidant. The optimized catalyst, Rb- Fe/CeO_2_, achieved complete aniline conversion (100%) with 91% selectivity toward azoxybenzene. The incorporation of Rb significantly improved the catalyst's electron transfer capability, reduced activation energy, and induced lattice distortion, leading to the formation of oxygen vacancies and Ce^3+^ species key contributors to the enhanced catalytic activity. Additionally, Rb modification helped suppress strong Brønsted acid sites, stabilized the Fe/CeO_2_ structure, improved Fe dispersion, and created an electron-rich environment that facilitated substrate activation. These synergistic effects increased the basic strength and electron density of the active Fe species, compensating for their inherent electronic limitations and resulting in improved catalytic efficiency. Reaction parameters such as catalyst loading (20–100 mg), reaction time (2–24 h), temperature (25–100 °C), solvent type, and volume (0.5–2 mL) were systematically studied in a 50 mL round-bottom flask with reflux conditions. DFT calculation (Fig. 9) has been done to determine interactions between substrate and catalyst surface.^2^

## 3 Conclusions

In conclusion, this comprehensive review highlights the significant advancements and challenges in the catalytic conversion of aniline into azo compounds. The development of efficient and sustainable catalytic systems has shown great promise in improving product yields, selectivity, and reducing environmental impact. Various catalysts, including homogeneous and heterogeneous systems, have been explored, and their mechanisms and performance have been discussed. Despite the progress made, further research is needed to overcome the existing limitations, such as catalyst stability and recyclability. Future studies should focus on designing novel catalysts, developing solvent-free protocols and methods that fully integrate green oxidants, optimizing reaction conditions, and investigating the underlying mechanisms to achieve more efficient and environmentally friendly processes. This review provides a foundation for researchers and industry professionals to advance the field of catalytic aniline conversion and develop sustainable methods for producing valuable azo compounds.

## Author contributions

The manuscript was written through contributions of all authors. All authors have given approval to the final version of the manuscript.

## Conflicts of interest

The authors declare no competing financial interest.

## Funding

This work was supported by the Deanship of Scientific Research, Vice Presidency for Graduate Studies and Scientific Research, King Faisal University, Saudi Arabia [grant no. KFU252772].

## Data Availability

Data is available on request.
